# Autologous mitochondrial transplantation enhances the bioenergetics of auditory cells and mitigates cell loss induced by H_2_O_2_

**DOI:** 10.1016/j.mito.2024.102003

**Published:** 2024-12-25

**Authors:** Mustafa Nazir Okur, Adam Ratajczak, Arash Kheradvar, Hamid R Djalilian

**Affiliations:** a Department of Otolaryngology-Head and Neck Surgery, School of Medicine, University of California Irvine, Irvine, CA 92697, USA; b Department of Biomedical Engineering, University of California, 2420 Engineering Hall, Irvine, CA,92697-2730, USA; c Department of Neurological Surgery, School of Medicine, University of California Irvine, Irvine, CA 92697, USA

**Keywords:** Mitochondrial transplantation, Hearing loss, Mitochondrial dysfunction

## Abstract

Hearing loss is a widespread and disabling condition with no current cure, underscoring the urgent need for new therapeutic approaches for treatment and prevention. A recent mitochondrial therapy approach by introducing exogenous mitochondria to the cells has shown promising results in mitigating mitochondria-related disorders. Despite the essential role of mitochondria in hearing, this novel strategy has not yet been tested for the treatment of hearing loss. More importantly, whether cochlear cells take up exogenous mitochondria and its consequence on cell bioenergetics has never been tested before. Here, we showed that exogenous mitochondria from HEI-OC1 auditory cells internalize into a new set of HEI-OC1 cells through co-incubation in a dose-dependent manner without inducing toxicity. We observed that auditory cells that received exogenous mitochondria exhibited increased bioenergetics compared to the controls that received none. Furthermore, we found that mitochondrial transplantation protects cells from oxidative stress and H_2_O_2_-induced apoptosis, while partially restoring bioenergetics diminished by H_2_O_2_ exposure. These findings support initial evidence for the feasibility and potential advantages of mitochondrial therapy in auditory cells. If successful in animal models and ultimately in humans, this novel therapy offers prominent potential for the treatment of sensorineural hearing loss.

## 1. Introduction

Hearing loss poses a significant health challenge, impacting millions of individuals across the United States and imposing a considerable economic and social burden (Emmett and Francis, 2015). Left untreated, hearing loss can lead to diminished social interactions and quality of life, often leading to depression and dementia in many individuals (Theunissen et al., 2011; Thomson et al., 2017; Tseng et al., 2018). Despite its widespread impact, there is currently no long-lasting treatment for hearing loss.

Hearing loss typically stems from progressive defects in key cochlear regions, particularly sensorineural structures such as sensory outer hair cells (OHCs) and inner hair cells (IHCs) (Raphael, 2002). These structures, rich in mitochondria and metabolically active, are susceptible to mitochondrial dysfunction, particularly when triggered by external factors such as loud noises or ototoxic drugs (De La Luz and Sordo, 2019; Henderson et al., 2006; Kamogashira et al., 2017, 2015; Okur and Djalilian, 2022; Puschner and Schacht, 1997; Tan and Song, 2023). Currently, various studies and clinical trials focused on enhancing mitochondrial function utilize pharmaceutical compounds or nutritional supplements to address hearing loss (Feng et al., 2020; Fujimoto and Yamasoba, 2019; Okur et al., 2023; Okur and Djalilian, 2022; Wu et al., 2019). However, these drug-based therapies adversely target mitochondrial regulators that intersect with other vital biological pathways, leading to an inadvertent cascade of unforeseen outcomes (Pitceathly et al., 2021; Vasan and Chandel, 2024; Vodičková et al., 2022). Indeed, many of these agents failed in clinical trials for the treatment of hearing loss, emphasizing the need for novel therapeutic strategies (Pitceathly et al., 2021; Vasan and Chandel, 2024; Vodičková et al., 2022).

A cutting-edge approach known as mitochondrial therapy or mito-therapy, involving the transplantation of healthy mitochondria into damaged organs such as the heart, has recently shown promising results in mitigating mitochondrial dysfunction (Hosseinian et al., 2022; Liu et al., 2022; Pour et al., 2021). For example, the preliminary success in both efficacy and safety of mito-therapy in animal models experiencing lung reperfusion injury has shown a potential to treat the most serious conditions that currently lead to high mortality due to a lack of effective therapies (Moskowitzova et al., 2020). The protective effects of mitochondrial transplantation have also been observed in retinal cells, which exhibit morphological similarities to sensory cells in the cochlea, given their shared origin from sensory epithelia (Nascimento-dos-Santos et al., 2020). Some studies have shown that transplanted mitochondria into the retina safeguard cells from optic nerve injury up to 4 weeks post-transplantation (Nascimento-dos-Santos et al., 2020). Notably, this approach was conducted in a pilot study involving pediatric patients, with no safety concerns detected (Guariento et al., 2021). At the cellular level, transplanted mitochondria are observed to function within various types of recipient cells, stimulating oxygen consumption, increasing ATP production, and enhancing cell survival and proliferation (Kitani et al., 2014; Pacak et al., 2015). Whether cochlear cells uptake exogenous mitochondria and its potential physiological implications remain unexplored and despite the crucial role of mitochondria in hearing (Okur and Djalilian, 2022; Tan and Song, 2023), this novel strategy has yet to be tested for the treatment of hearing loss. In this study, through a series of *in vitro* experiments, we investigated the feasibility of transplanting mitochondria into cochlear cells, assessing potential toxicity, and testing whether transplanted mitochondria enhance cellular bioenergetics while potentially offering protection against H_2_O_2_-induced damage.

## 2. Methods

### 2.1. Cell culture

We utilized mouse HEI-OC1 cells as the recipient auditory cells given that these auditory cells are considered as a progenitor for sensory and supporting cells of the organ of Corti and they express specific markers of cochlear hair cells (Kalinec Paul Webster David J Lim Federico Kalinec et al., 2003). We received HEI-OC1 cells from Dr. Federico Kalinec, and cultured those in Dulbecco’s modified Eagle medium (DMEM) (Corning, #10–013-CV) supplemented with 10 % fetal bovine serum (FBS) (ATCC, #30–2020) under permissive conditions (10 % CO_2_ at 33 °C) in a humidified incubator, according to their maintenance protocols described in a prior publication (Kalinec et al., 2016).

### 2.2. Mitochondrial isolation

Mitochondria were isolated from mouse HEI-OC1 using a commercially available isolation kit for cultured cells (Thermo Fisher Scientific, #89874). A fraction of isolated mitochondria (2.5 % of total isolated mitochondria) was lysed in RIPA buffer (Cell Signalling, #9806) to measure concentration using Pierce^™^ BCA Protein Assay Kit (Thermo Fisher Scientific, #23227).

### 2.3. Mitochondrial transplantation and imaging

To facilitate the approach, isolated mitochondria were co-incubated with HEI-OC1 cells for 24 h in complete media (10 % FBS + DMEM). Cells in the control group were solely cultured in complete media.

**Fig. 1.**
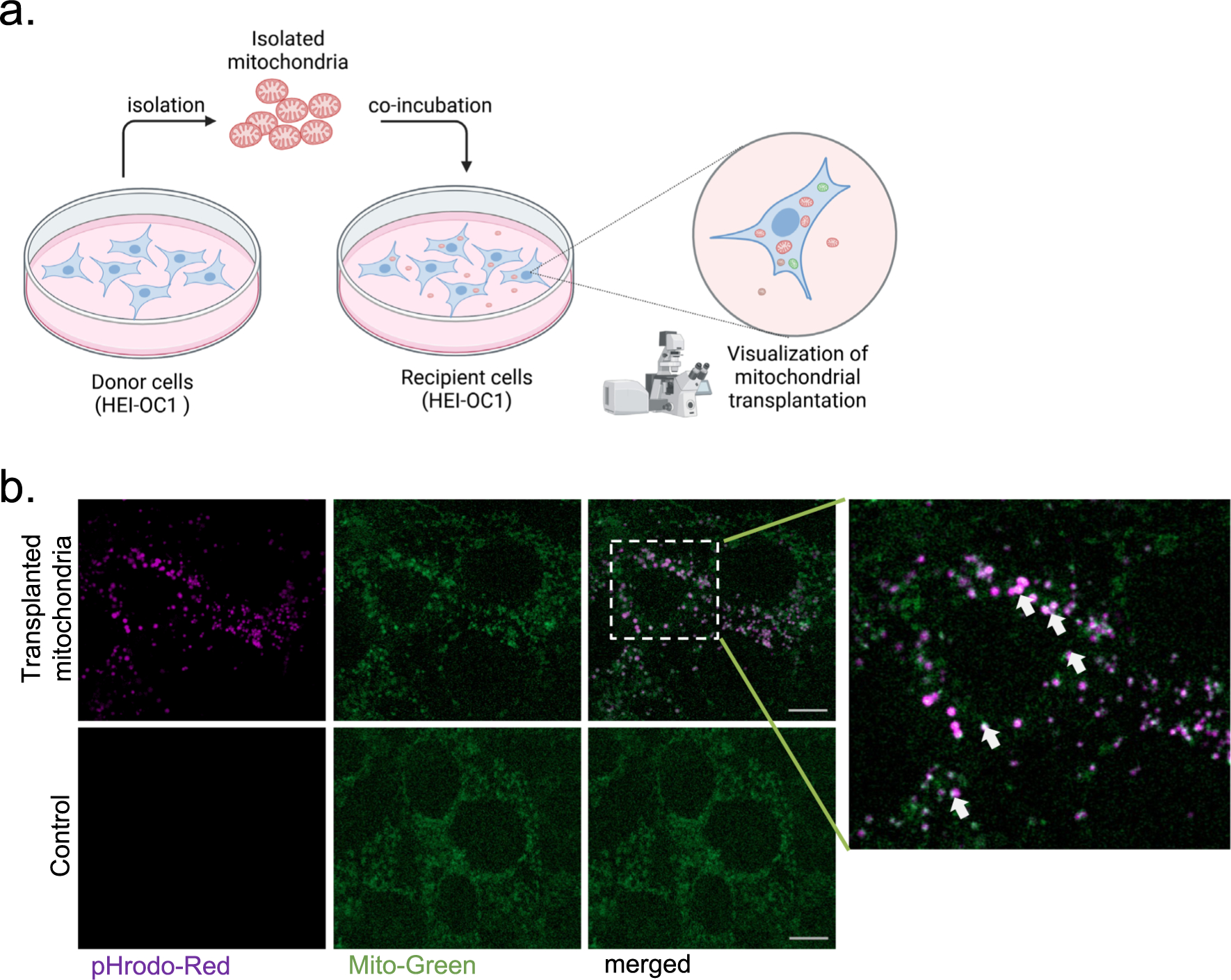
Exogenous mitochondria internalize into auditory (HEI-OC1) cells. **a.** The diagram illustrating the process of mitochondrial transplantation in cultured auditory cells. **b.** 2.5 μg of exogenous mitochondria isolated from mouse HEI-OC1 cells are labeled with pHrodo Red Succinimidyl Ester and co-incubated with a new set of HEI-OC1 cells (10 K) for 24H. The green staining represents native mitochondria labeled with mitoTracker green, while the violet staining indicates transplanted mitochondria labeled with pHrodo-Red. The scale bar represents 10 μm. White arrows point to the colocalization of native and exogenous mitochondria.

For visualization purposes, 20,000 HEI-OC1 cells (or as specified in the figure panels) were plated on a μ-slide 8-well ibiTreat (ibidi, #80826) one day prior to mitochondrial transplantation. They were then stained with MitoTracker Green FM (Thermo Fisher Scientific, # M7514) at 33 °C for 45 min before co-incubation. Meanwhile, isolated exogenous mitochondria were stained with pHrodo Red Succinimidyl Ester (Thermo Fisher Scientific, # P36600) for 30 min at 4 °C, followed by two washes with sterile PBS. Finally, HEI-OC1 cells and isolated mitochondria were co-incubated at 33 °C for 24 h. The pHrodo Red dye are non-fluorescent outside the cell and only fluorescent when absorbed within live cells (Pour et al., 2020). Following co-incubation, mitochondrial transplantation into cells was visualized using confocal microscopy (Zeiss LSM 900 Airyscan 2) as shown in Fig. 1. As a negative control to ensure that pHrodo^™^-Red dye does not independently diffuse into the cells, excessive amounts of pHrodo^™^-Red (1ug/ml) without exogenous mitochondria were incubated with cells. Images were analyzed via Fiji ImageJ software (NIH, Bethesda, MD).

### 2.4. Assessment of HEI-OC1 Cells’ bioenergetics

Oxygen consumption rate (OCR) measurements in HEI-OC1 cells were performed using a Seahorse XF24 flux analyzer (Seahorse Bioscience/Agilent, Santa Clara, CA). To identify the optimal cell-seeding density for measuring OCR, HEI-OC1 cells were first seeded on a 96-well plate at various densities ranging from 20,000 to 60,000 per well. Following experimentation, the optimal density was found to be at 40,000. Subsequently, 40,000 HEI-OC1 cells were plated into a Seahorse tissue culture plate overnight. The media was replaced the next day with Seahorse XF DMEM (Agilent Technologies) supplemented with glucose to a final concentration of 25 mM, sodium pyruvate to 10 mM (Gibco, 11360–070), and L-Glutamax (Gibco, 35050–061) to 2 mmol/L. The cells were incubated for 1 h at 33 °C under ambient oxygen and carbon dioxide concentrations prior to beginning of the measurements. OCR measured at sequential treatment with 2 μM for oligomycin, 0.6 μM for carbonyl cyanide-p-trifluoromethoxyphenylhydrazone (FCCP), and 2 μmol/L for rotenone + 2 μmol/L of antimycin A (R + A). Immediately after the measurements, cells were lysed using RIPA buffer (Cell Signalling, #9806), and the protein content was assessed using Pierce^™^ BCA Protein Assay Kit (Thermo Fisher Scientific, #23227). Afterwards, the respiration rates for each well were normalized to their respective protein concentrations.

### 2.5. H2O2 treatment

The complete media were removed from the cells and replaced with DMEM containing 100 μM H_2_O_2_ (VWR, #MK524002) for one hour unless indicated otherwise in the figure panels. The control group was treated solely with DMEM. Following the treatment, the DMEM media with or without H_2_O_2_ were replaced with complete media.

### 2.6. Cell viability and growth assay

After 3,000 HEI-OC1 cells were seeded on a 96-well plate and were incubated overnight, on the next day (considered as Day 0), cells were counted using a C-Chip disposable hemacytometer (Bulldogbio, #DHC-N01) and immediately co-incubated with various amounts of isolated mitochondria for 24H. The growth characteristics of the cells were observed and recorded every 24 h. The growth curve was generated by normalizing the initial cell counts on Day 0. A trypan blue exclusion test was conducted to assess cell viability (Strober, 2015). In summary, the cells at 72 h were washed with PBS, detached from the well via trypsinization (0.05w/v% trypsin, 0.53 mM EDTA, for 2 mins, 33 °C), and then diluted (1:1) in 0.4 % trypan blue solution. The cells taking up or excluding trypan blue dye were counted using a C-Chip disposable hemacytometer.

### 2.7. Cellular ROS detection assay

Intracellular ROS levels in auditory cells were measured using the DCFDA Cellular ROS Detection Assay Kit (Abcam, #ab113851), according to the manufacturer’s instructions. HEI-OC1 cells (25,000 per well) were seeded into 96-well microplates and incubated for 24 h. Cells were treated with DMEM containing 100 μM H_2_O_2_ for 1 h, while the control group received DMEM alone. After treatment, the DMEM (with or without H_2_O_2_) was replaced with complete media containing 5 μg of exogenous mitochondria. Following 24 h of co-incubation, cells were washed with PBS, and DCFDA working solution was added. The cells were incubated in the dark at 33 °C for 45 min, followed by a second PBS wash. Fluorescence intensity was then measured using the BioTek Cytation 5 Cell Imaging Multimode Reader (excitation/emission = 485/535 nm) and normalized to the negative control.

### 2.8. Apoptosis detection assay

Apoptotic rates in auditory cells were assessed using the CellEvent^™^ Caspase-3/7 Green ReadyProbes^™^ assay (ThermoFisher, #R37111) according to the manufacturer’s protocol. Briefly, 25,000 HEI-OC1 cells were seeded per well in 96-well microplates and incubated for 24 h. The cells were then treated with DMEM containing 100 μM H_2_O_2_ for 1 h, while the control group was treated with DMEM alone. After treatment, the DMEM (with or without H_2_O_2_) was replaced with complete media containing 5 μg of exogenous mitochondria. Following 24 h of co-incubation, 40 μL/mL of CellEvent^™^ Caspase-3/7 Green ReadyProbes^™^ reagent was added to each well, and the cells were incubated in the dark at 33 °C for 30 min. Fluorescence signals indicative of Caspase-3/7 activation were measured using the BioTek Cytation 5 Cell Imaging Multimode Reader (excitation/emission = 502/530 nm). Fluorescence intensity was then normalized to the negative control.

### 2.9. Statistical analyses

A two-tailed *t*-test was used to assess variations between the two groups. A one-way ANOVA with Tukey’s post hoc test was applied to detect significant distinctions among multiple samples while a two-way ANOVA with Tukey’s post hoc test was employed across multiple samples with two groups unless indicated otherwise in the figure panels. All statistical analyses were carried out using GraphPad Prism version 7 (GraphPad Software, Inc.).

## 3. Results

### 3.1. Internalization of exogenous mitochondria into auditory (HEI-OC1) cells through co-incubation

We first isolated mitochondria from auditory HEI-OC1 cells (donor) and co-incubated them with a new population of HEI-OC1 cells (recipient) as shown in Fig. 1a. To distinguish between the two mitochondrial populations, before co-incubation, we labeled the donor mitochondria with pHrodo^™^-Red SE (violet) and the endogenous mitochondria in recipient cells with MitoTracker Green FM (green). We selected pHrodo^™^-Red SE because it selectively stains mitochondria and fluoresces only within live cells, without passively diffusing into cells on its own (see Methods for details) (Pour et al., 2020). After 24 h of co-incubation, we observed that the exogenous mitochondria (violet) had successfully internalized into the recipient HEI-OC1 cells (Fig. 1b, top panels). In contrast, cells treated only with pHrodo^™^-Red SE dye (1 μg/ml) displayed no fluorescence (Fig. 1b, bottom panels), confirming that the dye does not passively enter cells without active mitochondrial uptake to carry it into the cellular environment.

### 3.2. Internalization of exogenous mitochondria into the auditory (HEI-OC1) cells

**Fig. 2.**
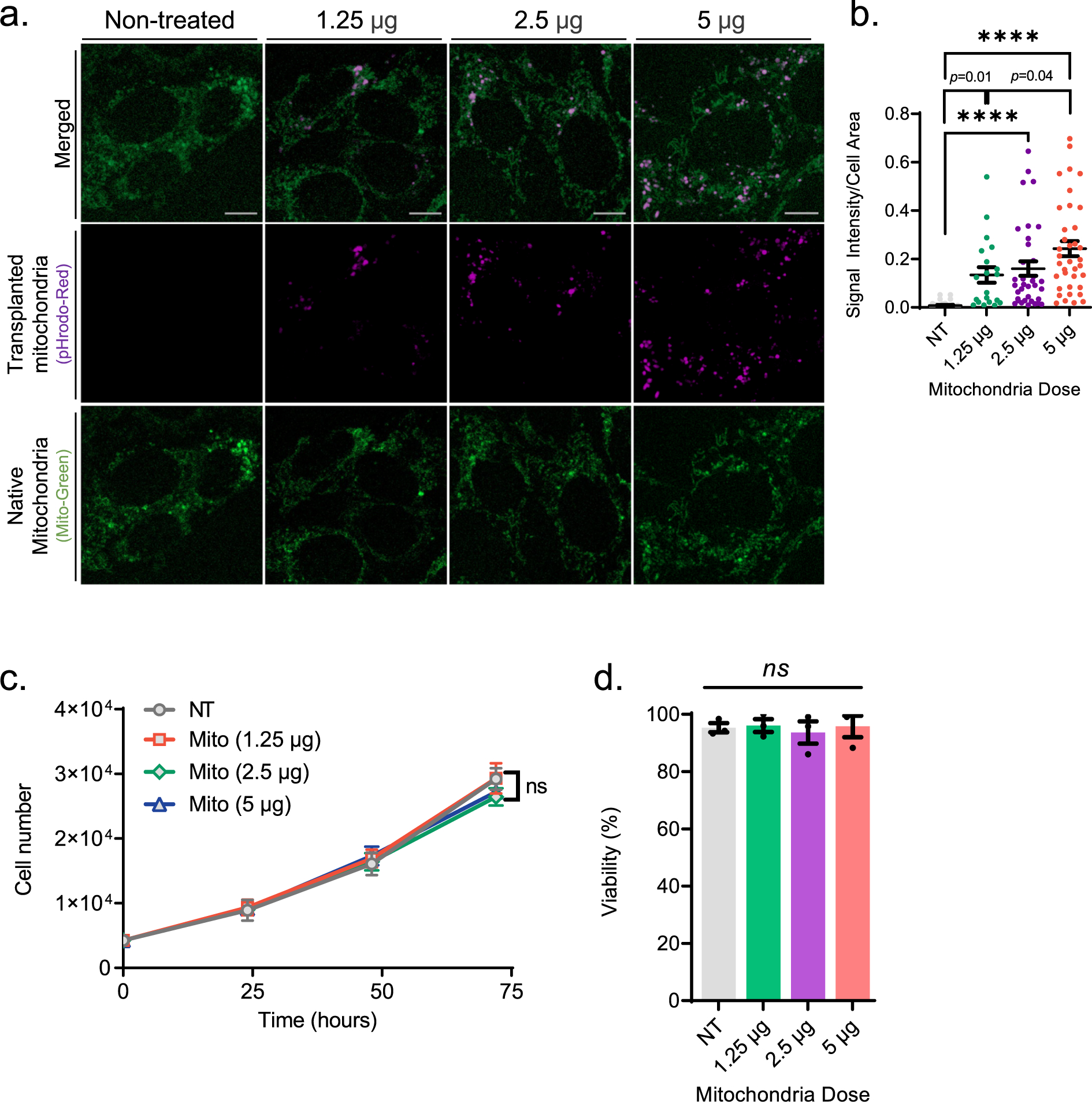
Exogenous mitochondria internalize into auditory (HEI-OC1) cells in a dose-dependent manner without inducing toxicity. **a.** Representative confocal images depict the transplantation of exogenous mitochondria (1.25 μg, 2.5 μg, and 5 μg) at various dosages into HEI-OC1 cells (10 K) via 24H co-incubation. The green staining represents native mitochondria labeled with mitoTracker green, while the violet staining indicates transplanted mitochondria labeled with pHrodo Red SE. **b.** The graph demonstrates the average fluorescence intensity of pHrodo Red SE per cell area in (a). The internalization of transplanted exogenous mitochondria (isolated from HEI-OC1 cells) into a new set of HEI-OC1 cells (10 K) is demonstrated to be dose-dependent (**b**) without significantly affecting cell viability (**c**). data represented as mean ± SEM, *ns* (not significant); ****p < 0,0001.

After confirming a successful transplantation, we tested whether this phenomenon is dose-dependent. To address this, we co-incubated HEI-OC1 cells with exogenous mitochondria at various concentrations (1.25 μg, 2.5 μg and 5 μg) and found that mitochondrial uptake indeed increases with escalating doses of exogenous mitochondria (Fig. 2a and 2b). Next, we tested whether transplanted mitochondria have any adverse impact on HEI-OC1 cells. To do so, we treated cells with various amounts of exogenous mitochondria similar to those above and assessed cell growth and viability. As shown in Fig. 2c and 2d, we found that mitochondrial treatment neither adversely affects the doubling rate nor viability, even at the highest dose tested (5 μg). This data suggests that exogenous mitochondria internalize into auditory cells in a dose-dependent manner without inducing any detectable toxicity.

### 3.3. The exogenous mitochondria prevent H_2_O_2_-induced decline in cell growth

**Fig. 3.**
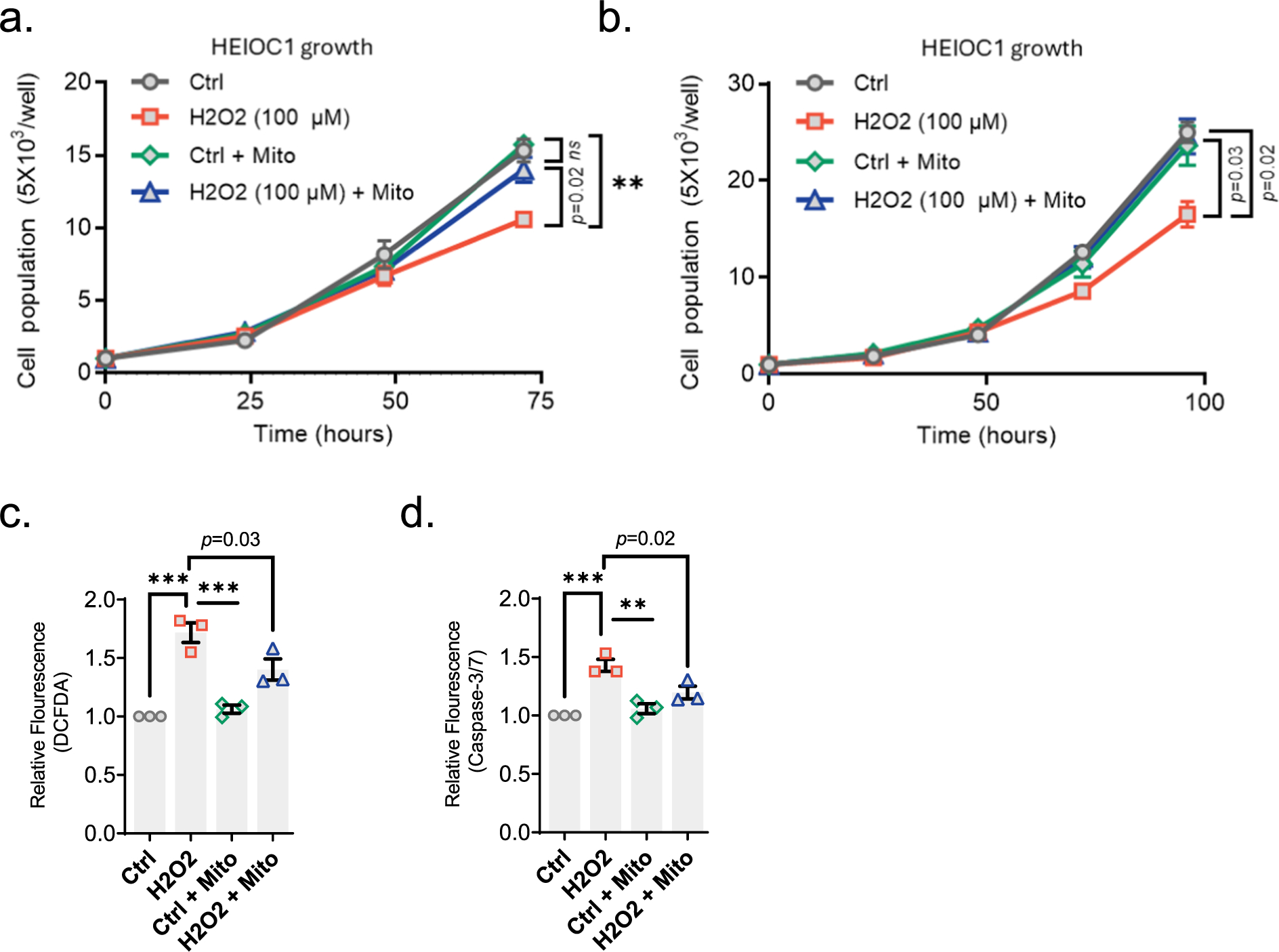
Mitochondrial Transplantation Protects Auditory Cells from Oxidative Stress and Apoptotic Death Induced by H_2_O_2_. a. Following exposure to H_2_O_2_ (100 μM) for 1 h, HEI-OC1 cells were treated with exogenous mitochondria (5 μg, extracted from HEI-OC1 cells). Subsequently, the assessment of cell growth was conducted. b. Following co-incubation with exogenous mitochondria (5 μg extracted from HEI-OC1 cells), HEI-OC1 cells underwent exposure to H_2_O_2_ (100 μM) for a duration of 1 h. Subsequently, the assessment of cell growth was conducted. Data represented as mean ± SEM, n = 3, *ns* (not significant). c-d. Following exposure to H_2_O_2_ (100 μM) for a duration of 1 h, HEI-OC1 cells were treated with exogenous mitochondria (5 μg, extracted from HEI-OC1 cells) for 24 h. cellular ROS levels (DFCDA) (c) and apoptotic rate (caspase 3/7 activation) (d) were measured. DFCDA (2′,7′ –dichlorofluorescein diacetate), data represented as mean ± SEM, n = 3, *ns* (not significant); ** p < 0.01; *** p < 0.001.

Next, we investigated the effect of mitochondrial transplantation on auditory cells that are under oxidative stress. To do this, we initially treated HEI-OC1 cells with exogenous mitochondria (5 μg) for 24 h, then exposed them to the increasing doses of oxidizing agent H_2_O_2_ (100, 250, and 500 μM) for 1 h and subsequently evaluated the cell growth. We observed that exposure to H_2_O_2_ significantly reduced the growth rate in non-treated cells (Fig. 3a and Supp. Fig. 1a–b). The decline in growth rate was significantly restored with mitochondrial treatment (*p* = 0.02) in cells treated with 100 μM of H_2_O_2_ (Fig. 3a), while no effect of treatment was observed with higher H_2_O_2_ doses (250 and 500 μM) (Supp. Fig. 1a–b). Next, we investigated whether transplantation of mitochondria improves cell growth following exposure to H_2_O_2_. To do this, we initially exposed HEI-OC1 cells to H_2_O_2_ (100 μM, 1 h), followed by treatment with exogenous mitochondria (5 μg, 24 h), and subsequently assessed cell growth. Similarly, H_2_O_2_ exposure significantly reduced the growth rate of HEI-OC1 cells in all doses tested. Mitochondrial transplantation rescued the decline in growth rate induced by a low dose of H_2_O_2_ exposure (100 μM) (*p* = 0.02) (Fig. 3b) while no significant effect of treatment was observed at higher doses (250 and 500 μM) (Supp. Fig. 1c–d).

### 3.4. Mitochondrial transplantation mitigates H_2_O_2_-induced oxidative stress and apoptotic cell death in auditory cells

H_2_O_2_ exposure causes cellular damage in auditory cells, as elevated ROS levels disrupt cellular homeostasis and trigger pathways leading to apoptotic death (Li-Yang et al., 2024). Therefore, we next tested if the protective effect of mitochondrial transplantation against H_2_O_2_ exposure is mediated through its impact on reducing oxidative stress and apoptosis. To do this, we exposed HEI-OC1 auditory cells to H_2_O_2_ to induce oxidative stress, transplanted mitochondria, and measured cellular ROS levels. Consistent with previous studies (Li-Yang et al., 2024), we observed that H_2_O_2_ exposure elevates ROS levels in HEI-OC1 cells. However, mitochondrial transplantation significantly reduces these elevated ROS levels (*p* = 0.03), indicating its effectiveness in mitigating oxidative stress. Next, we assessed the impact of mitochondrial transplantation on H_2_O_2_-induced apoptotic cell death. We found that H_2_O_2_ exposure increases the apoptotic rate in auditory cells while this increase was significantly attenuated by mitochondrial transplantation (*p* = 0.02).

### 3.5. Transplantation of exogenous mitochondria enhances cell bioenergetics and partially restores the H_2_O_2_-induced decline in auditory cell bioenergetics

**Fig. 4.**
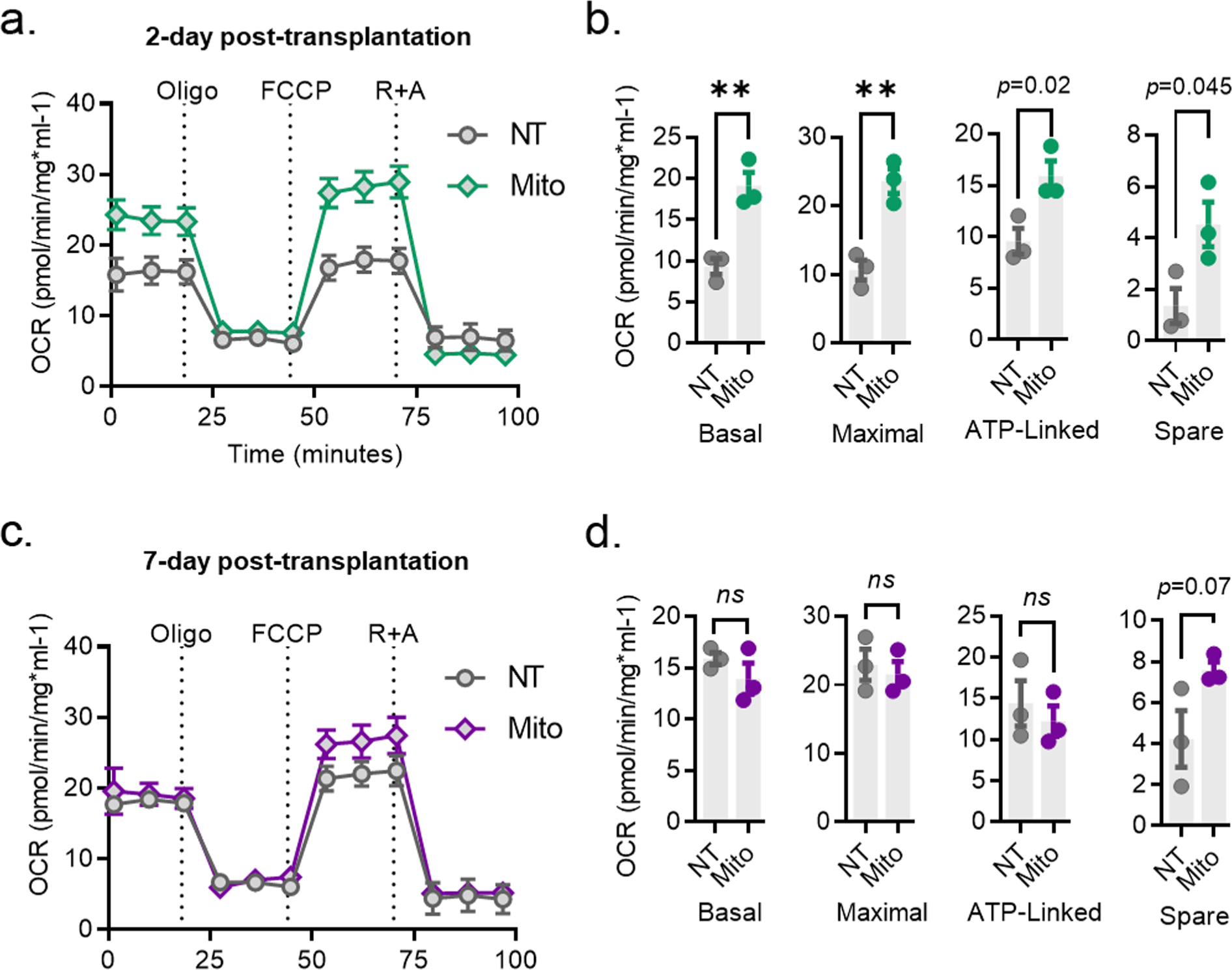
Bioenergetics consequences of mitochondrial transplantation in auditory cells in 2- and 7-days post-transplantation. Exogenous autologous mitochondria (5 μg, isolated from HEI-OC1 cells) were co-incubated with a new set of HEI-OC1 cells for 24H and OCR, basal respiration, maximal respiration, ATP-linked respiration, and reserve capacity were measured at 2 days post-transplantation (**a-b**) or 7 days post-transplantation (**c-d**) via a Seahorse XF24 flux analyzer. Oligo (oligomycin), FCCP (carbonyl cyanide-p-trifluoromethoxyphenyl hydrazone), R + A (rotenone and antimycin A), data represented as mean ± SEM, n = 3, *ns* (not significant); ** p < 0.01.

Our next effort was to examine whether mitochondrial transplantation confers any benefits on the bioenergetics of recipient cells. To investigate, we assessed the oxygen consumption rate (OCR) using an Agilent Seahorse Extracellular Flux Analyzer in cells treated with mitochondria (5 μg, 24 h) compared to non-treated cells. We found that transplantation of mitochondria into HEI-OC1 cells leads to an improvement in OCR and a significant increase in basal respiration (*p* < 0.01), maximal respiration (*p* <0.01), ATP-linked respiration (*p* =0.02), and spare capacity (*p* = 0.045) within 2 days post-transplantation (Fig. 4a and 4b). We then explored whether the improvement in bioenergetics mediated by transplantation remains sustained over time. Therefore, we evaluated the bioenergetics of auditory cells over a longer term (7 days post-transplantation). We observed no significant difference in bioenergetic parameters (OCR, basal respiration (*p* = 0.07), maximal respiration (*p* = 0.66), ATP-linked respiration (*p* = 0.54)) but only a trend of improvement in spare capacity (*p* = 0.07) in auditory cells at 7 days post-transplantation (Fig. 4c and 4d).

**Fig. 5.**
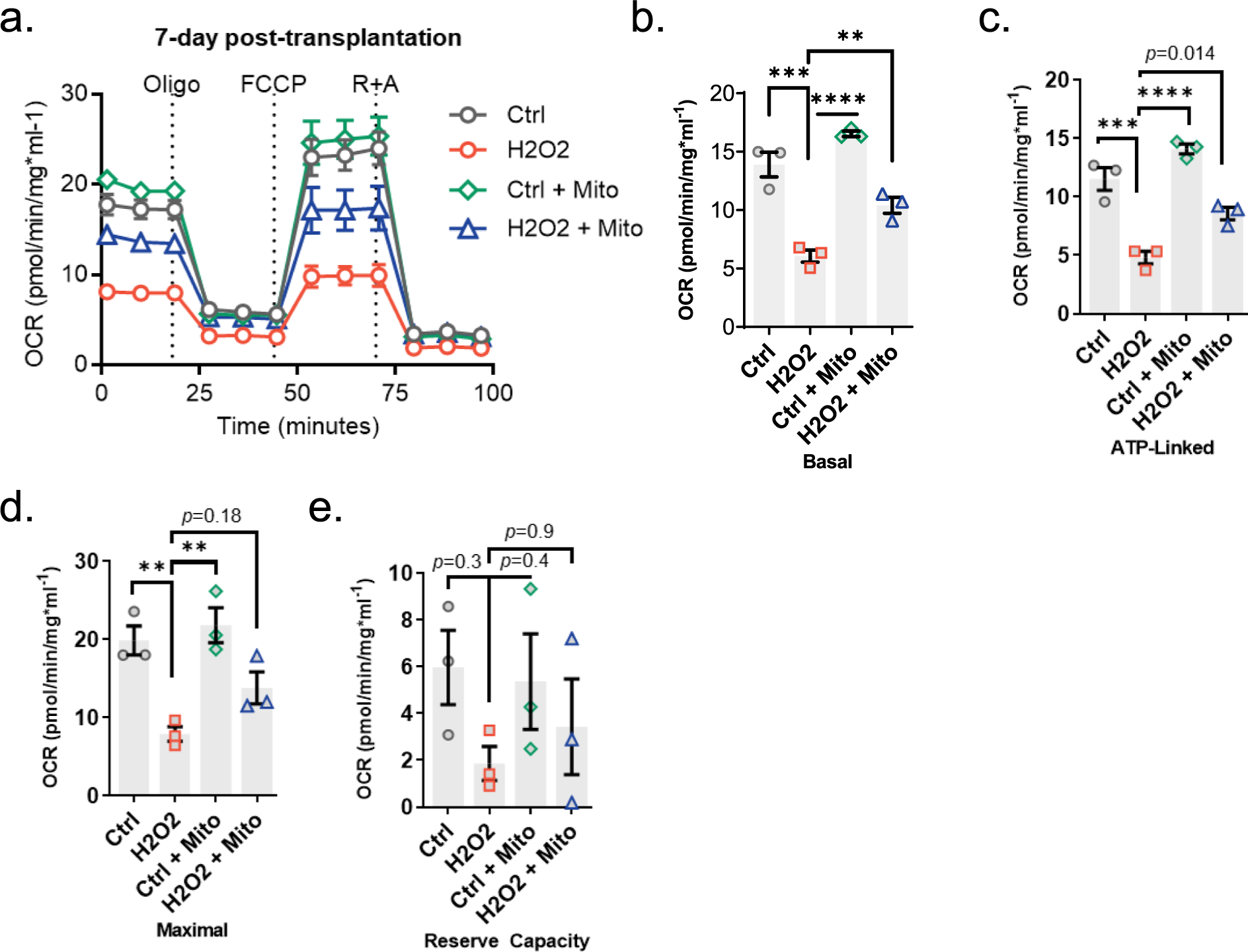
Autologous mitochondrial transplantation partially reinstates the decline in bioenergetics of auditory cells induced by H_2_O_2_. **a-b.** HEI-OC1 cells were exposed to H_2_ O_2_ (1H, 100 μM), followed by co-incubation with exogenous mitochondria (5 μg, isolated from HEI-OC1 cells) for 24H. OCR, basal respiration, maximal respiration, ATP-linked respiration, and reserve capacity were measured at 7 days post-transplantation by using the Seahorse XF24 flux analyzer. Abbreviations used: Oligo (oligomycin), FCCP (carbonyl cyanide-p-trifluoromethoxyphenyl hydrazone), R + A (rotenone and antimycin A), data represented as mean ± SEM, n = 3, *ns* (not significant); **p < 0.01, ***p < 0.001, ****p < 0,0001.

Previous studies suggested that the presence of external stress enhances mitochondrial transplantation efficiency and improves the adaptation of transplanted mitochondria in cells (Lee et al., 2022; Masuzawa et al., 2013). Therefore, we tested whether external stress has an impact on the transplantation-mediated benefits over a longer term (7 days post-transplantation). To achieve this, we initially exposed HEI-OC1 cells to H_2_O_2_ (100 μM, 1 h), followed by treatment with exogenous mitochondria (5 μg, 24 h), and subsequently assessed bioenergetics at 7 days post-transplantation. We observed that H_2_O_2_ exposure lowers OCR, and significantly reduces basal respiration (*p* = 0.0002), ATP-linked respiration (*p* = 0.0004), and maximal respiration (*p* = 0.007) (Fig. 5 a–d) with no significant effect on reserve capacity (*p* = 0.37) in non-treated cells (Fig. 5e). Notably, we found that mitochondrial transplantation significantly restored H_2_O_2_-induced decline in basal (*p* = 0.009) and ATP-linked respiration (*p* = 0.014) (Fig. 5b and 4c) while no significant difference was observed in other bioenergetic parameters (proton-linked respiration [*p* = 0.24], maximal respiration [*p* = 0.18], and reserve capacity [*p* = 0.91]).

## 4. Conclusions

In this study, for the first time, we demonstrate successful autologous transplantation of exogenous mitochondria from HEI-OC1 auditory cells into a new set of HEI-OC1 cells via co-incubation (Fig. 1). We found that co-incubating recipient cells with increasing doses of exogenous autologous mitochondria leads to increased mitochondrial uptake by the recipient cells with no toxic effect on cell growth and viability (Fig. 2). The lack of toxicity, even in the presence of excessive doses of mitochondria, up to 5 μg, underscores the safety of mitochondrial transplantation as a potential future therapy for cochlear disease. Interestingly, we observed that some of the transplanted mitochondria co-localize with native mitochondria in the recipient cells (Fig. 1b, white arrows), suggesting the ability of exogenous mitochondria to fuse with the endogenous mitochondrial network.

Following successful transplantation, we carried on investigating whether transplanted mitochondria display any benefits to the recipient auditory cells. It is widely accepted that noise exposure or ototoxicity causes reactive oxygen species (ROS) overproduction in cochlear tissue, eventually leading to oxidative stress, damaging cochlear cells, and consequently resulting in cell death (Li-Yang et al., 2024; Tan and Song, 2023; Yuan et al., 2015). Remarkably, transplanted mitochondria promote resistance to oxidative stress and improve bioenergetics in various cell types (Li et al., 2014; Lin et al., 2022; Maeda et al., 2020; Pour et al., 2020). We thus tested whether these benefits of transplantation also apply to auditory cells under stress. Our results indicate that autologous mitochondrial transplantation effectively prevents H_2_O_2_-induced increases in cellular ROS levels and protects auditory cells from apoptosis caused by H_2_O_2_ exposure (Fig. 3c and d). These protective effects were also evident in cell proliferation, as mitochondrial transplantation successfully counteracted the growth decline observed in cells treated with low doses of H2O2 (100 μM) (Fig. 3a). Remarkably, the benefits of mitochondrial transplantation on cell growth were evident both before and following H_2_O_2_ exposure (Fig. 3b), suggesting that transplantation not only enhances resistance to oxidative damage but also facilitates recovery from such damage. At higher doses of H_2_O_2_ (250 μM and 500 μM), however, mitochondrial transplantation did not rescue cell growth, indicating that the protective effects of mitochondrial transplantation may have a threshold beyond which it cannot counteract extensive oxidative damage.

Our experiments also led to a new understanding that mitochondrial transplantation acutely boosts auditory cell bioenergetics, including reserve capacity, basal respiration, maximal respiration, and ATP-linked respiration (Fig. 4a and 4b). These observations suggest that autologous mitochondrial transplantation elevates the abundance of mitochondria within the cells while the transplanted mitochondria retain their function. Additionally, we observed that the aforementioned enhancements returned to levels similar to those of the baseline control 7 days post-transplantation, suggesting that the acute improvement in auditory cell bioenergetics gradually diminishes over time (Fig. 4c and 4d). A similar phenomenon was also observed across various cell types by other researchers. For example, rat retinal ganglion cells exhibited heightened spare capacity 24 h after mitochondrial transplantation, yet this enhancement diminished by the seventh day (Nascimento-dos-Santos et al., 2020). Similarly, in rat cardiomyocytes, the initial improvements in basal, maximal, and ATP-linked respiration dissipated by the seventh-day post-transplantation (Pour et al., 2020). Given these results, one can speculate that healthy cells might adjust to the presence of non-native mitochondria by recalibrating their mitochondrial dynamics. This adaptation may involve degradation of their mitochondria content or adjustments in fusion and fission mechanisms. Eventually, these cells may restore their bioenergetic functions to baseline levels over time, especially in the absence of a need for additional energy expenditure. This is a topic that is worth further investigation.

Previous studies showed that the beneficial effect of mitochondrial transfer on energy metabolism lasts longer (at least 28 days) in ischemic cardiomyocytes (Masuzawa et al., 2013) vs. healthy cardiomyocytes (Pour et al., 2020), suggesting that external stress may improve the adaptation of transplanted mitochondria. In line with these findings, we observed that mitochondrial transplantation partially restores a decline in cell bioenergetics in H_2_O_2_-treated cells even on the seventh day post-transplantation while no significant effect was observed on untreated healthy cells (Fig. 5). This phenomenon is suspected to arise from oxidative stress induced by H_2_O_2_, which eventually damages both cells and their mitochondria. Consequently, there is an escalation in energy expenditure to repair the harm, coupled with a decline in energy production due to damaged mitochondria. As a result, cells may retain non-native mitochondria for a longer period to compensate for the reduced energy production capacity. Additional investigation is warranted to elucidate this mechanism.

**Fig. 6.**
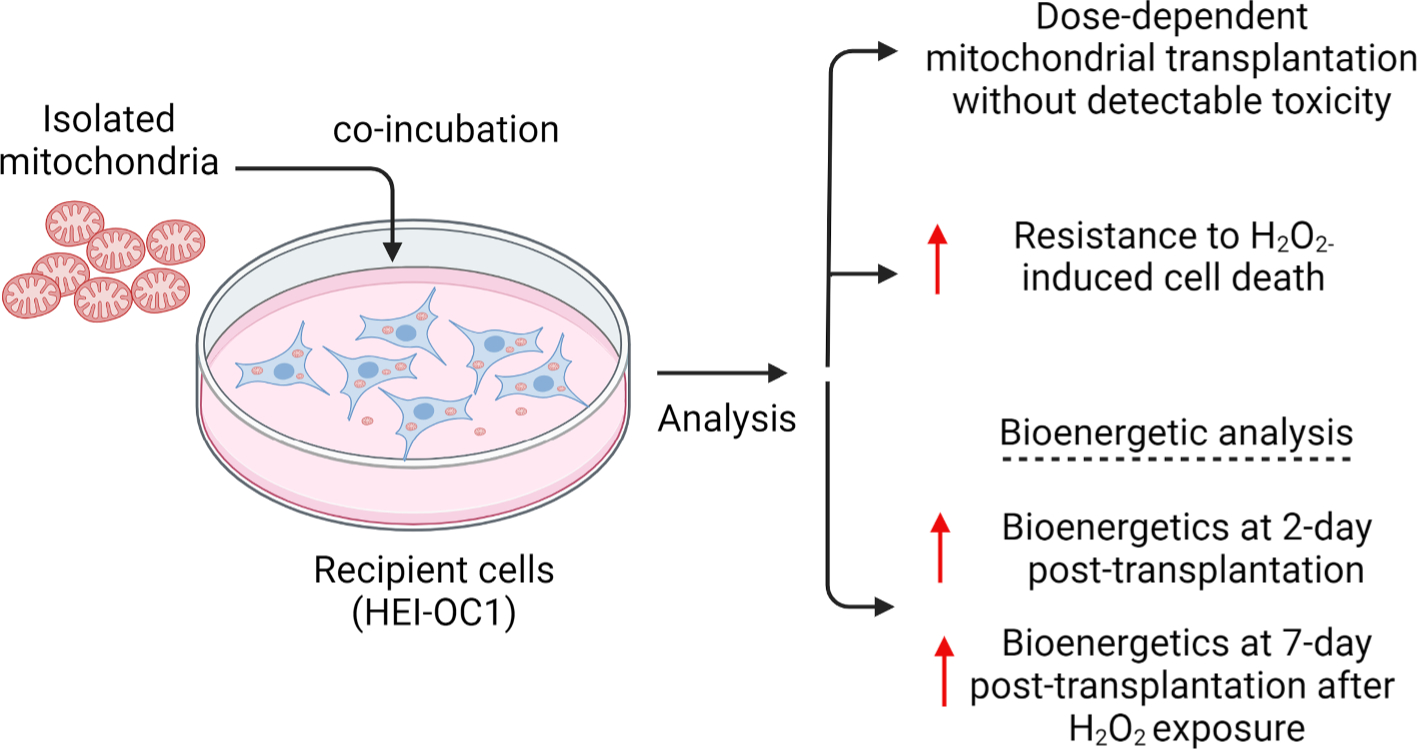
Autologous mitochondrial transplantation improves the bioenergetics of auditory cells and reduces H_2_O_2_-induced cell loss without inducing any detectible toxicity.

In summary, our findings indicate that autologous mitochondrial transplantation boosts acute bioenergetics of auditory cells and enhances their ability to withstand oxidative stress (Fig. 6). These findings are particularly relevant for conditions like noise-induced hearing loss, ototoxicity, and age-related hearing decline, where oxidative stress is a known pathological factor. Future studies should investigate the molecular mechanisms underlying the protective effects of mitochondrial transplantation, including its impact on mitochondrial dynamics, ROS-scavenging enzymes, and apoptotic pathways. Importantly, we observed no adverse effects on cell growth, viability, or intermediate-term bioenergetics, supporting the safety of mito-therapy for auditory cells (Fig. 6). This study marks a significant step forward in exploring autologous mitochondrial transplantation as a potential therapeutic approach for preventing or treating certain forms of hearing loss.

## Supplementary Material

Appendix A. Supplementary material

## Data Availability

Data will be made available on request.
