# Supplementary material for: Autologous mitochondrial transplantation enhances the bioenergetics of auditory cells and mitigates cell loss induced by H2O2
Source: Mitochondrion. Author manuscript; Available in PMC 2025 May 20. (PMC12090353; doi:10.1016/j.mito.2024.102003)
Supplement: Appendix A. Supplementary material [file NIHMS2064711-supplement-Appendix_A__Supplementary_material.pptx]

## Slide 1
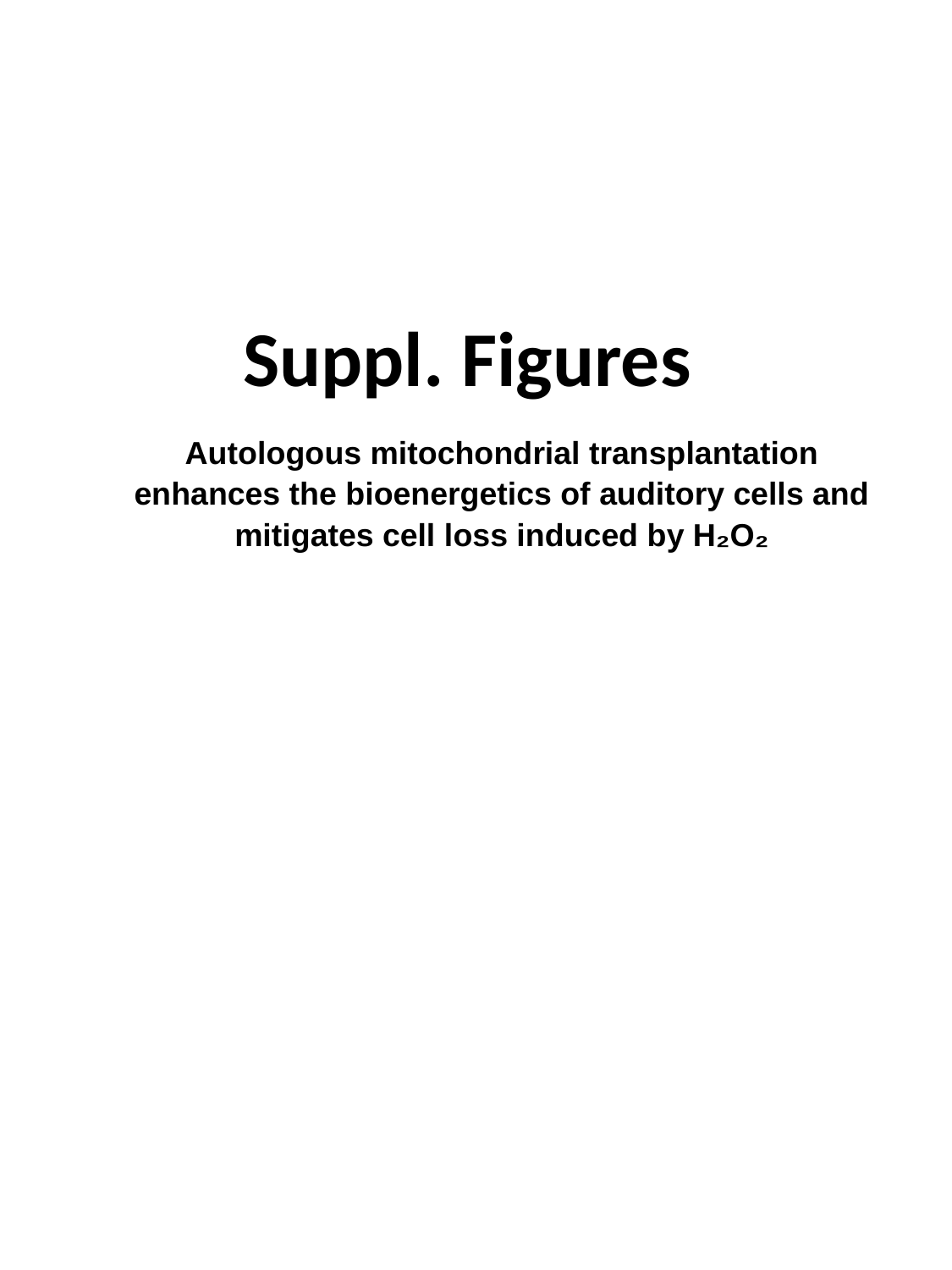

Suppl. Figures
# Autologous mitochondrial transplantation enhances the bioenergetics of auditory cells and mitigates cell loss induced by H₂O₂

## Slide 2
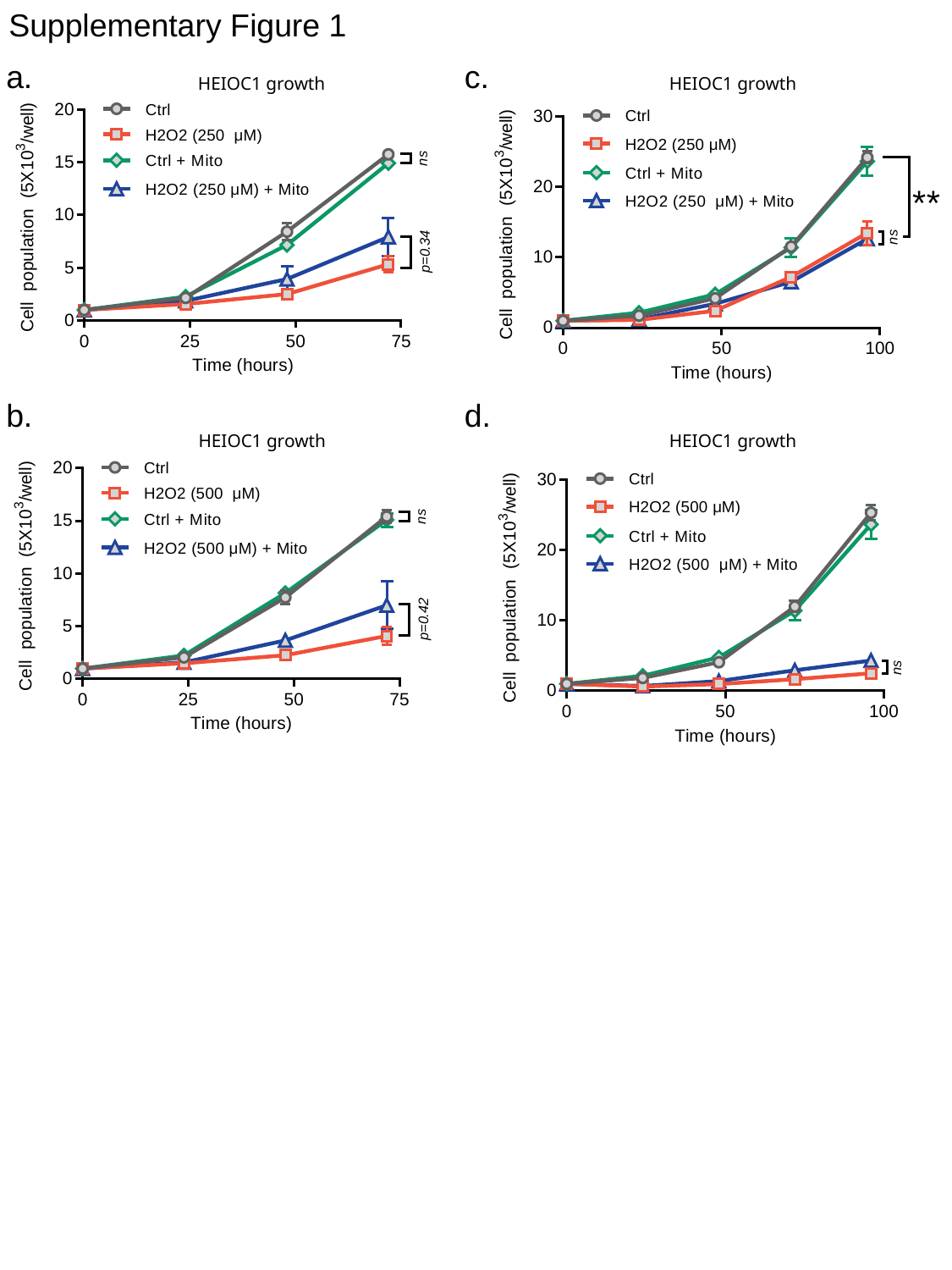

Supplementary Figure 1
a.
c.
HEIOC1 growth
HEIOC1 growth
b.
d.
HEIOC1 growth
HEIOC1 growth
